# Examining the role of macrolides and host immunity in combatting filarial parasites

**DOI:** 10.1186/s13071-017-2116-6

**Published:** 2017-04-14

**Authors:** Doug S. Carithers

**Affiliations:** Boehringer Ingelheim, 3239 Satellite Boulevard, Duluth, GA 30096 USA

**Keywords:** Avermectin, *Brugia malayi*, *Brugia timori*, *Dirofilaria immitis*, Macrocyclic lactones, Excretory-secretory apparatus, ES proteins, Filarial worms, Immunity, Ivermectin, Macrolides, Milbemycin, *Onchocerca cervicalis*, *Onchocerca volvulus*, *Wuchereria bancrofti*

## Abstract

Macrocyclic lactones (MLs), specifically the avermectins and milbemycins, are known for their effectiveness against a broad spectrum of disease-causing nematodes and arthropods in humans and animals. In most nematodes, drugs in this class induce paralysis, resulting in starvation, impaired ability to remain associated with their anatomical environment, and death of all life stages. Initially, this was also thought to be the ML mode of action against filarial nematodes, but researchers have not been able to validate these characteristic effects of immobilization/starvation of MLs in vitro, even at higher doses than are possible in vivo. Relatively recently, ML receptor sites exclusively located proximate to the excretory-secretory (ES) apparatus were identified in *Brugia malayi* microfilaria and an ML-induced suppression of secretory protein release by *B. malayi* microfilariae was demonstrated in vitro. It is hypothesized here that suppression of these ES proteins prevents the filarial worm from interfering with the host’s complement cascade, reducing the ability of the parasite to evade the immune system. Live microfilariae and/or larvae, thus exposed, are attacked and presented to the host’s innate immune mechanisms and are ultimately killed by the immune response, not the ML drug. These live, exposed filarial worms stimulate development of innate, cellular and humoral immune responses that when properly stimulated, are capable of clearing all larvae or microfilariae present in the host, regardless of their individual sensitivity to MLs. Additional research in this area can be expected to improve our understanding of the relationships among filarial worms, MLs, and the host immune system, which likely would have implications in filarial disease management in humans and animals.

## Background

Macrocyclic lactones (MLs), also known as macrolides, affect various life stages of many nematode species by acting primarily upon binding of a group of glutamate-gated chloride channels (GluCls), causing general locomotor and pharyngeal paralysis [1–4]. Although MLs (avermectins and milbemycins) are highly effective in treating microfilariae and preventing filarial infection, researchers have not been able to validate a clinically relevant immobilization/starvation effect of MLs on the filarial nematodes in vitro. This would indicate that a different mechanism of action is responsible for filarial clearance.

In 2010, Yovany Moreno et al. [5], identified ML receptor sites (a subunit of AVR-14 glutamate gated chloride channels, or GluCls) in microfilariae of *Brugia malayi*, one of the parasites responsible for lymphatic filariasis in humans. These ML receptor sites are located exclusively in the structures proximate to the excretory-secretory (ES) apparatus, which is the main source of microfilarial protein release. ES proteins have long been recognized for their immunomodulatory properties [6], including the ability to affect complement-fixing activity [7], allowing parasites to evade the host’s innate immune system. Researchers further demonstrated an ML-induced suppression of secretory proteins release by the microfilariae in vitro [5].

These reported bench observations, combined with this author’s clinical experience and observations, prompted an extensive literature review. The review supports the hypothesis that ML administration appears to limit ES protein release from the ES apparatus in microfilariae and juvenile filariae, which allows the host’s innate immune response recognize the microfilariae and larval worms as foreign entities. Additionally, the literature demonstrates that this immune recognition can generate a systemic host immune response capable of clearing microfilariae, or creating larval immunity, affecting new filarial infections. As with vaccination, the level of immunization conferred will vary with the individual, but typically depends on several factors: antigen level and location at the time of exposure, previous exposure, and the frequency and interval between subsequent exposures. So, a single ML administration is highly effective in causing exposure, and innate immune stimulation, resulting in clearance of affected larval and microfilarial stages of filarial worms. This exposure of the living larvae and microfilariae lends to potential development of both cellular and humoral immune responses, rather than humoral alone. If properly timed to allow for development of a memory response, strategic ML-dosing can be used to essentially booster systemic immunity, resulting in an immune response capable of killing all microfilarial or larval filariae, regardless of their ML sensitivity.

## Overview of filarial disease in humans and animals

Filarial nematodes are capable of causing significant disease with long-term ramifications in humans and animals. Intermediate hosts, or vectors, are involved in all instances. Interestingly, although they infect different locations in their respective hosts, the filarial worm species are remarkably similar in many ways.

In humans, the most prevalent filarial infections can result in blindness (*Onchocerca volvulus*) or lymphedema (*Brugia malayi*, *B. timori* and *Wuchereria bancrofti*).

The intermediate hosts of *O. volvulus* are blackflies, which transfer infective larvae to a susceptible person approximately 2 to 3 weeks after becoming infected by microfilariae in a blood meal from an infected host. The infective larvae migrate into the subcutaneous tissue in the competent host and form nodules under the surface of the skin while maturing into adult worms. Adult female *O. volvulus* produce 750 to 1600 microfilariae daily [8, 9]. When adult worms or microfilariae die, the resulting inflammatory response can lead to skin rashes; eye lesions, including corneal opacity; intense itching; and skin depigmentation [9].

Mosquitoes are the intermediate hosts for *Brugia* spp. and *Wuchereria*. Adults reside in the tissues of the lymphatic system, producing thousands to tens of thousands of microfilariae daily [10, 11]. In competent mosquito hosts, ingested microfilariae undergo larval development to become infective L3 that then can be transferred to humans and other susceptible mammals. In newly infected or reinfected humans, development to the adult stage is completed largely in the lymphatic system, with adult worms typically localized in lymph glands. Host reactions to the adult worms of *Brugia* spp. or *Wuchereria* can cause damage to the lymphatic system, affecting lymph flow, resulting in lymphedema or, in severe cases, elephantiasis [12].

Midges are the intermediate host of *Onchocerca cervicalis*. In horses*,* the *O. cervicalis* adults reside in the area of the nuchal ligament in the host horse’s neck, and microfilariae are distributed throughout the horse’s blood, lymph system, and tissue. As is the case in humans, the highest tissue concentrations of microfilariae are localized in the soft tissue near the gravid female.

Numerous mosquito species can serve as intermediate hosts for heartworms, *Dirofilaria immitis* in dogs. Following ingestion of *D. immitis* microfilariae from an infected host, the microfilariae migrate from the mosquito’s mid-gut to the malpighian tubules and develop into first-stage (L1) larvae, molt to second-stage larvae (L2), and then molt again into L3. The larvae become infective during their migration from the mosquito’s abdomen to its proboscis. When the mosquito feeds, larvae erupt through the tip of the labrum, emerging with a bit of hemolymph onto the surface of the skin. When the mosquito finishes feeding and withdraws its stylet, infective L3 migrate into the wound and molt to fourth-stage larvae (L4) in about 3–4 days in the subcutaneous tissue. L3 and L4 larvae are the only mammalian stages of *D. immitis* residing exclusively in the soft tissue of the host, generally a canid; all other life stages of *D. immitis* exist in the bloodstream.

The L4s continue growing for the next 45–65 days as they move through subcutaneous tissue and between muscle fibers. During the next phase of development, L4s molt and become immature adults, penetrate muscle tissue and eventually veins, and arrive at the heart and lungs, some as early as 67 days post-infection. When young adult heartworms enter the vascular system and reach the lungs, the flow of blood forces the heartworms into the small pulmonary arteries. As the worms grow, they extend into larger pulmonary arteries, becoming fully mature, breed, and begin to produce microfilariae. Each adult female heartworm is capable of producing more than 11,000 microfilariae per day. Microfilariae remain in the bloodstream, circulating for up to 2.5 years and awaiting ingestion by a competent intermediate host mosquito [13].

In dogs, heartworm infections cause significant pulmonary arterial and associated lung pathology, typically with related permanent sequelae, even after adult worms are eliminated by either medication or time. Note that humans can become infected with *D. immitis,* in which case the larvae seem to follow the same development pattern as in the canine host, ending up in the lungs where they die in small arteries, causing granulomatous lesions that resemble cancerous masses (“coin lesions”) radiographically. Excision biopsies of these coin lesions are necessary to differentiate *D. immitis* granulomas from lung cancer [14].

While the filarial parasites described here may seem vastly different since they affect different hosts and different organ systems, many aspects of these parasites and the respective host responses are extremely similar. The similarities of these filarial parasites can be seen in their individual familial responses, and lack of response, to MLs, in both in vitro and in vivo situations.

## Filarial nematodes and macrocyclic lactones

As noted, avermectins and milbemycins are effective against a wide range of nematode and arthropod parasites [15] and it is well documented that microfilariae and larvae are eliminated rapidly when MLs are administered to infected mammals [16–21]. ML anthelmintics act by binding to GluCls of nematodes and arthropods, causing these channels to open slowly but essentially irreversibly; leading to a long-lasting hyperpolarization or depolarization of the neuron, or muscle cell; and blocking further function [22]. Elimination of most species of nematodes is the consequence of ML activation causing general locomotor and pharyngeal paralysis [1–4].

Researchers assumed that similar paralyses occur in filarial worms [22]. Elimination of microfilarial and larval stages of filarial worms has been shown to occur rapidly in vivo following administration of low (< 10 μg/kg) or high (50–200 μg/kg) doses of MLs to infected individuals [16–18], and it has been demonstrated that ML targets exist in the filarial worm *D. immitis* [21, 22]; however, interestingly, adult stages of filarial worms are not killed in vivo by single or even multiple high doses of MLs [23–28].

It is important to note that the effects of MLs in vivo against microfilariae and larvae, as well as the lack of readily apparent effects on adults, are similar for filarial worms whether they infect humans (*B. malayi* and *Brugia pahangi* [29]; *Wuchereria bancrofti* [29]; *O. volvulus* [24]; and *Litomosoides carinii* [29]); dogs (*D. immitis* [16]; *Dirofilaria repens* [29]; and *Acanthocheilonema reconditum* [29]); horses (*O. cervicalis* [17]); cattle (*O. linealis* [18] and *O. ochengi* [28]); or rats (*Acanthocheilonema viteae* [29]).

The apparent inability of MLs to kill adult filarial worms appears to be at odds with the rapid in vivo efficacy of MLs against microfilariae and larvae, but this lack of clinically meaningful effects against adult stages has appeared to been consistent in vivo and in vitro in most species of filarial worms [18, 30–32]. As recently as 2011 investigators reported that variability in motor paralysis was observed in vitro in separate isolates of *D. immitis* microfilariae exposed to MLs [33], however the doses necessary to do so were so high (~70 μM) that any paralysis was likely an off-target effect (i.e. activation of non-target GABA - gamma-aminobutyric acid - sites). Paralysis was not validated, as Vatta et al. [34] found that complete paralysis of *D. immitis*, indicating microfilarial death, was not achievable in vitro at any tested ML dose. They noted the in vitro ML concentrations used to elicit what previous investigators described as ‘paralyses’ far exceeded ML doses safe for healthy, uninfected dogs. In fact, even when Vatta et al. [34] incubated microfilariae in 10 μM ivermectin for 16 h, paralysis remained incomplete. That dose is 217 times higher than the peak serum concentration in dogs receiving a 100 μg/kg oral dose of ivermectin - a dose known to be highly microfilaricidal in dogs [35]. The 10 μM microfilariae moved more slowly than those incubated at lower concentrations, but all were still motile.

Considering that MLs act as almost irreversible [36] long-acting agonists of susceptible GluCls [22, 37, 38], lack of in vitro activity suggests that filarial death is not caused primarily by a general locomotor and/or pharyngeal paralysis, as is the case with most nematodes. The differences between in vivo and in vitro results of ML administration in filarial worms versus other nematode species led Bennett et al. [39] to comment: “This raises the possibility that IVM (ivermectin) does not act directly on the parasite, but rather through synergism with the host immune system.” Since that time, a direct effect of IVM on filarial worms has been documented (i.e. decreased ES production) [5]. Therefore, while MLs do not potentiate a host immune response, it does appear that an interaction with the host immune system is necessary to eliminate microfilariae and larvae that are exposed to MLs. This literature review supports and provides further insight into this observation.

## The ES apparatus, ES proteins, and effects of ML administration on microfilariae

Although they are potentially long-lived [13], microfilariae are morphologically simple, which serves to make singling out and identifying anatomical components and the physiologic activity of those components relatively straightforward. Microfilariae are pre-larval stages of filarial parasites, persisting in the same form for days to years without changing, but developing rapidly through three larval stages once ingested by a competent intermediate host.

Microfilariae acquire nutrients across the cuticle, as they do not have a functioning gut; instead they have a gut thread, which will develop into a gut after microfilariae infect an intermediate host and develop through their larval stages [40]. Microfilariae have only two excretory organ systems, one ridding the worm of metabolic wastes and the other consistently producing specific proteins. Their pseudocoel is under pressure, which gives the parasites their roundworm shape, but this same structural pressure keeps both excretory pores closed, hence all excretory products produced by microfilariae must be expressed forcibly. The ES apparatus is the primary site for protein secretion [5], releasing what are referred to as ES proteins.

The ES apparatus is present in all life stages of most filarial parasites and remains a source for protein secretion throughout the life-cycle. ES protein production begins early in filarial worm development. Although not present in unfertilized ova, ES proteins are produced and present in developing eggs shortly after fertilization [41].

As noted, research on the filarial nematode *B. malayi* [5] using immunofluorescent staining of a known ML target (a subunit of AVR-14 glutamate-gated chloride channels) demonstrated the presence of these receptor channels in the subject microfilariae. Investigators validated this finding by demonstrating a specific, a direct, and quantifiable in vitro effect on ES production when microfilariae were exposed to low concentrations of ivermectin: exposure of microfilariae to ivermectin resulted in an apparent decrease in ES protein release by the microfilariae.

Thus, in vitro, minute concentrations of ML cause an apparent paralysis of the ES apparatus [5] in susceptible individual worms, leading to reduced ES protein production. It is hypothesized, here, that this observed reduction compromises the ability of microfilariae to affect complement fixation, thus exposing living microfilariae to the host innate immune response*.*

Staniunas & Hammerberg [42] noted that ES proteins allow local evasion of innate immune detection of *D. immitis* by affecting complement-fixing activity of the host. Interestingly, according to Hammerberg & Williams [7], many other tissue-dwelling parasitic organisms have been shown to produce remarkably similar complement-inhibiting proteins, and investigators have identified such proteins obtained from larvae in vitro from surface extracts and from cyst/vesicle fluid. Immunomodulating secretory proteins are found among the helminths: schistosomes [43]; nematodes: *Toxocara canis* [44] and the filarial nematodes *Setaria digitata* [41], *B. pahangi* [45], *W. bancrofti*, *O. volvulus* [46], *D. immitis* [42, 44, 45, 47]; tapeworms [48–50]; protozoans *Trypanosoma* [51, 52], *Leishmania* [53, 54]; amebae [55]; and even certain strains of bacteria (*Escherichia coli* and *Salmonella typhi* [56]), to name a few. In addition, the surface of trophoblast cells of mammalian embryos (including humans) produce proteins that have a similar effect on complement fixation and innate immunity, preventing maternal immune rejection of the fetus [57, 58].

Such complement-dependent triggering has been demonstrated in several in vitro studies, in which addition of MLs has facilitated the killing of different species of microfilariae by host cells, but only in the presence of serum [30, 34, 59], which indicates the need for complement in the process. Zahner & Schmidtchen [59] noted that in vitro, the addition of ivermectin affected but did not kill *L. carinii* microfilariae. However, when spleen cells of *Mastomys coucha* (Southern multimammate mouse) or rats were added, killing of microfilariae was induced. Similar observations were made by Vatta et al. [34], who noted adherence of peripheral blood mononuclear cells and neutrophils to microfilariae of *D. immitis* in vitro following exposure to ivermectin, but only in the presence of serum. They concluded that these results were consistent with a model in which MLs interfere with the parasite’s ability to evade the host’s innate immune system. This provides insight into the lack of in vitro ML effectiveness against microfilariae, but it does not explain all of the in vivo host-responses that affect filarial nematodes.

## ML administration to filarial worms

Since the discovery, development, and commercialization of MLs, there have been various theories about ML mode of action against filarial worms. Historically, two hypotheses have been predominant: MLs kill microfilariae and larvae directly, in which case in vitro activity would be expected to be readily observable; or MLs affect the uterus of adult female worms directly, causing microfilarial production to cease or be suspended for a period of time [60]. While some researchers hypothesized that ML activity is linked with host immunity in some way, or that MLs affect the guidance capabilities of filarial worms, typically MLs were regarded as responsible for killing the parasites.

Although the predominant hypotheses were not illogical, and some components might have merit, many aspects of ML activity remained unexplained: How could ivermectin, a drug that is essentially completely excreted within a week after administration [15, 35, 39, 61] affect microfilarial production of some species for up to 12 months or longer? In *D. immitis* infections in dogs, why won’t a single microfilaricidal dose sterilize the female worms as it typically does with *O. volvulus* infections in humans, or *O. cervicalis* in horses? How can repeated monthly low-dose MLs eliminate *D. immitis* microfilariae in dogs, when single high doses cannot? In human filarial infections, if the drug effect of MLs on the uterus of the female worms were direct, why is there such great variability in the duration of amicrofilaremia?

In situations where the ML response is less than optimal, as has been reported with onchocerciasis and heartworms, it has been theorized that reduced susceptibility is due to PGP efflux pump modifications and/or b-tubulin mutations. An alternate theory that this author might propose is there could simply be variation in the total number of ML-targeted receptor sites associated with the ES apparatus in individual larvae and microfilariae, and an alternate receptor is more responsible for ES vesicle expression. Whatever the ultimate cause or mechanism, the commonality is that a direct chemotherapeutic effect of MLs in a select population of individual filarial worms is incomplete. Fortunately, there are other means of attacking resistant filarial worms. Facilitated by well-timed ML-administration, immune memory responses can be generated that affect entire populations of microfilariae or larvae, in spite of each individual worm’s direct sensitivity to the effects of MLs.

### Natural microfilarial and larval antibody production

Johnson et al. [62] studied in vitro cellular adherence of eosinophils and neutrophils to microfilariae of *B. malayi* in cat serum. They found that adherence was mediated by both, heat-labile and heat-stable factors. The heat-labile factors, identified as complement components, caused adherence of cells to some older microfilariae but not to younger microfilariae. Investigators noted that the heat-stable factor, found in the serum of about 10% of the cats, were immunoglobulin G (IgG) antibodies. Unlike the compliment components, the IgG antibodies caused adherence equally in all ages of microfilariae, regardless of their effective levels of ES proteins.

Naturally occurring amicrofilaremic individuals have been documented in filarial-infected humans with *O. volvulus* [63], *W. bancrofti* [29, 64, 65], *B. malayi* [29, 62, 66], and *B. pahangi* [66], and in dogs infected with *D. immitis* [47, 67–70]. Wong & Suter [67] noted that when naturally amicrofilaremic *D. immitis-*infected dogs were treated to remove adult worms and then re-infected, the dogs were susceptible to subsequent infection, but the new infection was also amicrofilaremic. Weil et al. [68] reported that this is consistent with observations in other nematode and trematode systems, suggesting that in chronic tissue helminth infections there is suppression of cellular immune responses to parasite immunogens, while humoral responses to these same immunogens remain relatively well-preserved.

Mammalian hosts are also naturally capable of creating antibody responses to the surface proteins and secretory proteins of larval (L3 and L4) and adult stages of filarial worms [29, 65, 71–77]. These filarial proteins are presented to the host’s immune system through secretions, molting, or the deaths of developing larvae as they migrate through the tissue of the host, or adult worms wherever they reside. As in the case with antibodies against microfilariae affecting microfilariae, sufficient levels of antibodies against larvae will kill larvae, even if that larva is capable of inhibiting complement. However, as opposed to microfilarial immunity, larval immunity is capable of conferring protection, preventing development of adult worms in the putatively immune human or animal [29, 75, 78–83].

#### MLs and adult filarial worms

As noted, the hypothesis that MLs kill larvae and microfilariae directly led researchers to focus on the concept of direct activity against adult worms; however, an apparent lack of visible adulticidal activity in vitro and in short-duration in vivo studies resulted in a general decreased interest in continued exploration of potential adulticidal activity. Undaunted, a few researchers continued regular treatment scenarios out further and found long-term, pulse-dose administration of MLs to infected individuals affected and resulted in death of adult worms of *O. volvulus* [25, 27, 84] and *D. immitis* [85]. Steffens & McCall [86] noted changes in *D. immitis* ingesta and gut epithelium. According to the authors, the cells lining the gut were columnar, rather than the normal cuboidal; there were fewer intracellular mitochondria, which would reduce active transport; and there was an increase in intracellular lipids. This author believes the impact on adult viability that occurred in these longer duration studies could be due, at least in part, to an immunotherapeutic effect. What is being observed in the longer-term studies cited above could be the result of a memory response related to repeated pulse dosing of the infected individual. More studies will be needed to determine whether timely pulse dosing of MLs (boosting the immune response) over a period of time might offer more clinically effective activity against adult filarial worms in human hosts.

### Attempts to develop vaccines targeting filarial parasites

The ability of host immunity to ameliorate filarial infections has led numerous researchers to explore development of microfilarial and larval vaccines. The ultimate level of antibody production and cellular protection derived from any vaccine depends on several parameters, including vaccine type; the presentation site; the interval between challenges; antigen reactivity, that is, foreignness to the host and molecular size of the immunogen or immunogens presented by the pathogen; the dose volume and concentration of immunogen(s) in the vaccine; and the adjuvant, or immunostimulant, used. Researchers concede that a vaccine generating both humoral and cell-mediated immunity would be more effective [87, 88]. In the case of filarial nematodes, presentation of microfilarial and filarial larval antigens in the soft tissue via injection of irradiated larvae or abbreviated infection has been shown to elicit the most protective immune responses [67, 71, 78, 80, 89–93].

In order for subsequent vaccinations to boost an immune response effectively, a sufficient interval - typically at least 3 weeks - is required between doses to allow development of antigen-specific immune responses with minimal interference. If the exposure to challenge is continuous or if the interval is too short, immune tolerance, such as that generated in allergy desensitization, can result instead of immunization. Should the vaccine interval be too long, it will reduce the ability of the vaccinations to confer a strong or lasting immune response.

Even when a vaccine or vaccination protocol results in successful immunization, the resulting immune response and antibody levels will decline over time and eventually drop below protective thresholds, unless sufficient exposure to the same antigen reactivates immune memory. While historically the filarial vaccines elicited immune responses, they were typical of those for weakly immunogenic vaccines [94], and although a sterilizing vaccine could represent a “holy grail,” to date there is no commercialized antifilarial vaccine of any type [95, 96].

### Microfilarial and larval vaccination

#### Microfilarial vaccination

Theoretically, the concept of microfilarial vaccination is feasible; however the degree of success would depend on the specific filarial worm and host. Wong [97] demonstrated that uninfected dogs receiving several immunizing inoculations of *D. immitis* and *B. pahangi* microfilariae did not tolerate any circulating microfilariae, regardless of the extent of the challenge. When added to media containing living microfilariae in vitro*,* sera taken from these same vaccinated dogs agglutinated the living microfilariae. Ah et al. [98] observed a dramatic decline in *D. immitis* microfilarial counts following vaccination. Similarly, Wong & Suter [67] found that immunization with microfilariae caused some infected dogs to be amicrofilaremic throughout the span of the adult infection. They also found that other dogs developed adult infections that initially produced microfilariae and then became amicrofilaremic rapidly. This would indicate that in addition to the initial immunization, further immune stimulation from normal microfilarial attrition was needed in these individuals to achieve an antibody titer sufficient to clear the microfilariae. While, as expected, the level of antibody response generated by each individual test subject was not consistent, indirect fluorescent antibody test (IFA)-microfilarial titers were directly correlated to the level of amicrofilaremia; the higher the titer, the fewer microfilariae were present. Thus, investigators established that an antibody response is capable of reducing microfilarial burdens, potentially clearing them from the blood and tissue, maintaining amicrofilaremia for a period of time, and clearing subsequent challenges with microfilariae.

While high, antibody titers can be produced by vaccination, resulting in amicrofilaremia and affected developing microfilariae in the uterus of females, such titers do not prevent development of subsequent larval infections. This was observed in human filarial worms placed in competent animal models and filarial worms in animals with naturally occurring amicrofilaremia, as well as animals vaccinated with microfilarial-based vaccines [67, 78, 80, 97–100]. This phenomenon is explained by the fact that antibodies created in response to the microfilariae differ in many respects from antibodies generated by larval and adult stages; therefore microfilarial antibodies would not be expected to confer immunity that protects against a new larval infection [12, 91, 101–107].

#### Larval vaccination

Vaccination against larval infection is possible, as dose-dependent responses have been demonstrated using killed and irradiated microfilarial and larval vaccines of several filarial worm species, and abbreviated larval infections with *D. immitis* [67, 90, 92, 108], *O. volvulus* [109], *Loa loa* [110], *B. malayi* [71, 111] and *W. bancrofti* [112]. Additionally, concurrent administration of the immunostimulant Freund’s complete adjuvant (FCA) with an abbreviated larval infection elicited a stronger protective immune response than an abbreviated infection without FCA, or an irradiated infection alone [92].

For example, three abbreviated infections were used by Grieve et al. [90] to immunize dogs against subsequent *D. immitis* challenge. The dogs were infected with 150 to 400 *D. immitis* infective L3, each about 1 mm in length. Each infection was allowed to develop for 62 days before being abbreviated by administration of oral ivermectin. At that point, larvae would have molted at least once and some twice, depositing antigen from those molts in the soft tissue. Additionally, a small number of larvae would have been expected to die during migration, depositing antigen and perhaps priming an immune response. The researchers observed a 98% reduction in adult parasites at 7 months post challenge in immunized dogs versus controls.

After having been allowed to develop for 62 days, the larvae would have grown to an average of approximately 10 mm in length and 0.1 mm in diameter [113]. Thus, delaying treatment until day 62 resulted in a higher larval antigenic mass being presented in the host. This would be expected to elicit a stronger local immune response than would have been generated had infective larvae been cleared immediately upon infection.

In these and previous abbreviated infection vaccine studies, the assumption was that the microfilariae and larvae were killed by the ML, so any immune response generated was in response to dead filarial targets. Thus, the expectation was that any systemic response was due to antibody alone, or an antibody-dependent cell-mediated cytotoxicity system [87]. Based upon our current understanding of ML activity described in this paper, this would not be the case. This understanding clearly explains what Folkard, et al. described as “unexpected results” with SCID mice in 1997 [88]. SCID mice are characterized by an absence of functional T cells and B cells, lymphopenia, hypogammaglobulinemia, and a normal hematopoietic microenvironment. According to the authors, SCID mice are unable to clear microfilariae in the skin, and they confirmed, using adoptive transfer of spleen cells, that clearance was dependent upon CD4^+^T cells and is associated with high levels of circulating IL-5. Unexpectedly, they found that in naïve SCID mice that were infected with microfilariae, then cleared with ivermectin were then highly resistant to reinfection with microfilariae even in the absence of T and B cells. These results were supported by Soboslay et al. who suggested that repeated treatment with ivermectin facilitated parasite-specific innate cellular response in onchocerciasis patients, and that this may reduce the serious morbidity of chronic *O. volvulus* infection [114]. Since ML treatment is capable of stimulating innate immune responses, as well as both antibody and cell mediated immune responses, this helps explain why the immune response generated far exceeds that of kill vaccines, and is similar to or better than that of irradiated larval vaccines.

In ascending order the most successful filarial vaccines tested thus far have utilized irradiated larvae, abbreviated infections, and then abbreviated infections with FCA; the search continues for delivery options and specific immunogenic proteins [96, 104, 105, 111, 115–117].

The microfilariae and filarial larvae exposed to the immune system tend to be reactive, but not extremely reactive, so individual host immune responses generated are apt to vary [67, 108, 118] and eventually wane. However, repeated doses at sufficient intervals with either live or killed vaccines elicit a memory response, which results in a higher antibody titer and more effective immune protection [93, 109, 110, 112].

### Filarial population density and the host-immune-system interaction

It seems clear that substantial vaccine-development challenges exist and that MLs will continue to play a significant role in controlling and/or preventing filarial disease. However, armed with a better understanding the activity of MLs against filarial worms, and the role of the host’s immune system in worm death should facilitate changes in how MLs are used. The focus should now shift to utilizing ML protocols that maximize the potential immune response, rather than relying on higher/longer-acting ML doses.

#### Tissue-dwelling filarial worms

Much of the microfilarial populations of *O. volvulus*, *B. malayi*, *B. timori* and *W. bancrofti* will disseminate throughout the circulatory or lymphatic systems, but with a patent infection the highest localized concentration of microfilariae will be the newly produced microfilariae in the tissue immediately proximate to the adult worms. Following ML administration, these pockets of concentrated microfilariae in tissue present to the immune system suddenly. Thus, they would essentially act like a subcutaneous or intramuscular vaccination. Once recognizable by the innate immune system, cells adhere and attack microfilariae and a localized inflammation occurs. This mobilization leads to recognition, antibody production, and potentially development of cell-mediated immunity, much as with any live vaccine [119]. As with vaccines, the immune response is dependent in part upon the microfilarial burdens. MLs administered to individuals with higher burdens leading to a stronger antibody response, a more rapid clearance of microfilariae, and a longer duration of clearance due to affecting embryonic microfilariae in the uterus of the female worm [23, 25, 84]. The fact that *O. volvulus* adults are present in nodules under the skin allowed these authors to ascertain infection and further allows the opportunity to examine the adult worms. Interestingly, the same observations were recorded in adult female worms taken from treated individuals as from those collected from naturally amicrofilaremic infections, in which unfertilized, up to early trophoblast ova persisted in the uterus of female worms. The absence of later stages and microfilariae points occurring in both instances points to a phenomenon that is immunologic, rather than a direct ML-effect on the worm’s uterus. This conclusion is logical as ML drug would be eliminated rapidly, however treated individual hosts would remain amicrofilaremic for up to a year or even longer. Conversely, should the tissue-based microfilarial burden in an individual be lower (i.e. in newly patent female worms, or low burden infections), then the microfilarial burden would be expected to be less, and any immune response to ML-treatment would be weaker, resulting in a short duration of amicrofilaremia following treatment with MLs. Thus, instances of observed variability in the duration of amicrofilaremia following ML dosing within *Onchocerca*-endemic communities could have a much less sinister explanation than resistance.

As with human tissue filariasis, horses infected with the nuchal worm *O. cervicalis*, a single dose of an ML can result in long-term amicrofilaremia [17]. In individual horses, the post-ML immune response can result in a localized inflammatory response in the neck and generalized, severe dependent edema [120]. Note that individual humans infected with *Onchocerca* may also experience post-treatment edema, papular dermatitis, and an intense pruritus due to microfilarial clearance [9]. Similar durations of amicrofilaremia and reactions have been noted in ML-treated cattle infected with *O. linealis* [18] and *O. ochengi* [28].

#### Vascular-dwelling filarial worms

In individual dogs infected with *D. immitis*, a single dose of ML can lead to extended amicrofilaremia, but more typically, this is not the case. Since adult *D. immitis* reside in the pulmonary arteries and microfilariae are deposited into and reside in the bloodstream, the immune response to *D. immitis* microfilariae would be expected to be weaker; much weaker than immune responses seen for tissue-dwelling filarial infections in humans and animals. Thus, ML treatment of *D. immitis* positive dogs leads to dissemination of dying microfilariae in capillary beds throughout the body [19, 20] and no localized extravascular presentation of antigen, resulting in a diminished immune response. As a consequence, there is generally incomplete clearance or transient amicrofilaremia, with a rebound in microfilarial counts if no further ML dosing is administered [108]; however, subsequent, properly timed ML dosing elicits a booster effect with each dose, eventually resulting in sufficient antibody levels to cause amicrofilaremia even if adult worms are still present. Thus, a long-term effectiveness is likely due to an immunologic effect, potentiated by regular, episodic exposure of microfilariae caused by administration of the ML-drug. When dosing ceases, immune stimulation ceases, measured antibody levels wane, and microfilarial production might begin again [97, 108].

## Discussion

The author’s literature review supports the conjecture that exposure to MLs, causes susceptible juvenile stages of filarial worms to lose their ability to camouflage themselves from the host’s innate immune system. In fact, evidence that MLs rely on the host’s immune response to ‘exposed’ filarial worms is not surprising, as the concept that the ultimate activity of an anthelmintic drug is the result of an immunologic process is not new. Diethylcarbamazine (DEC) employs a different mechanism than that of MLs, but it still targets filarial worms by facilitating an enhanced stimulation of the innate immune response [42, 121–123].

Interpretation of the literature in light of recognizing that filarial worms are exposed to the immune system as a consequence of ML activity, all the ensuing events that occur can be explained by known immunological mechanisms and supported by previously reported data on vaccine-generated immune responses to filarial worms.

Historically, assumptions regarding ML mode of action against microfilariae and filarial larvae did not consider immunity as the significant factor causing filarial worm death, rather the immunologic response was thought to be a by-product of dead worms, rather than living worms. However, the research described in this review indicates that the protective immune response generated and demonstrated by use of MLs in abbreviated filarial infections more closely mirrors the response seen with irradiated microfilarial and larval vaccinations, which is similar to the response that would be expected to a modified live vaccination. This indicates ML administration presents live larvae to the immune system, conferring a more rapid, profound immune response than would result had the filarial worms been killed directly and immediately by the ML. Grieve et al. [93] noted this immunologic killing was multistage and affected larval growth, as well as causing larval killing 3 weeks after challenge. Interestingly, later Grieve [90] commented that this research suggested that immunogenic components associated with live worms - that is the total quantity or unique presentation of antigenic mass - constitute the common denominator for a successful vaccine.

### Timing ML-administration to maximize the host immune response

In light of these considerations, one may envision studies and reassessments undertaken by experts in human and animal parasitology and immunology. Such research and/or review could result in adjustments or redirection in disease prevention and treatment protocols and ongoing ML-related research.

#### Tissue-dwelling filariasis

Lymphatic filariasis programs in endemic areas worldwide have focused on control and elimination by monitoring and administering ivermectin or DEC together with albendazole once a year, or albendazole, alone, twice a year in locales where *Loa loa* is also present [124]. This approach can be expected to reduce microfilariae available for ingestion by the intermediate hosts, but only if all infected individuals were identified, had sufficient infections, and treated appropriately. Since the oral ML dose would be essentially excreted in less than 12 days, even a 2-dose/year ML protocol would only be minimally effective in preventing non-infected individuals from becoming infected, as acquired infective larvae would develop unabated. If an untreated source for infection of the intermediate host existed nearby, naïve individuals and those receiving treatment with an ML more than 2 weeks previously could acquire new infections.

If a program were designed to treat both infected and potentially exposed individuals, using more frequent or monthly dosing intervals, this approach could target newly acquired larval infections. The ML-driven exposure of larvae to the host immune system could allow the host to eliminate susceptible larvae, and repeated ML dosing could potentially stimulate the immune response should a challenge exist, preventing development of adult infections and potential lymphadenopathies. Instances of *Onchocerca cervicalis* in horses and *O. linealis* in cattle are now a rarity in the US, likely due to the regular use of MLs in these domesticated animals.

As is the case in lymphatic filariasis, the focus of eradication efforts has been on monitoring and administering anthelmintic medication to infected individuals at least once a year [125]. Since humans are the lone definitive hosts for *O. volvulus*, it may be preferable to treat all known infected individuals and prevent new infections in potentially exposed individuals with modified treatment intervals. Year-round treatment would be necessary infected/treated individuals as well as the uninfected population in a given endemic area, along with vector control. ML administration, followed by a microfilarial vaccine booster(s) could also be an option to consider in locales where resistance is a concern, or year-round monthly prevention is not possible.

Testing the viability of such management plans could be possible with a concentrated effort using geographically isolated pockets of infection. Should any protocol show promise, rapid reduction or elimination of infection potential in the intermediate host population and a resulting decreased risk for the human population could quickly be assessed.

It is important to acknowledge that these are conjectures, and that local conditions, resources, cultural practices, and ongoing, yet-to-be-published research could affect potential protocol changes, but these concepts remains worth considering in designing future studies and interpreting past study results.

#### Vascular-dwelling filarial worms

Keeping the more susceptible tissue-dwelling stages of *D. immitis* larvae from developing into adult worms in individual pet dogs is the objective of heartworm disease prevention, as countless wild, feral and untreated canines act as reservoir hosts. When dosed appropriately, MLs are highly effective in clearing infective *D. immitis* larvae in almost every infection. Regular ML dosing has been highly effective in preventing development of larval stages into adult infections, but once patent, the *D. immitis* stages residing in the bloodstream continue to present challenges related to genetic diversity.

In fact, in the infancy of MLs in the early 1980s, multiple doses of ivermectin were necessary to clear microfilarial infections in some infected dogs in clearance studies. Campbell [15] described a set of studies in which 96% of 121 dogs became negative following a single ivermectin dose of 50 μg/kg. The remaining 4% of dogs required several doses at monthly intervals to clear microfilariae, suggesting that not enough drug was used, or not all microfilariae were susceptible and that administration of multiple doses could have led to an immune response that cleared the remaining non-susceptible worms.

The effects of selection pressure in the *D. immitis* microfilarial population have been demonstrated in controlled studies that relied solely on the chemotherapeutic effect of MLs. Researchers used single high-dose MLs to elicit chemotherapeutic pressure on known infected and microfilaria-positive dogs. In these studies, within days following administration of a high-dose ML, available remaining microfilariae were collected and isolated in a laboratory colony, grown to infective larvae in mosquitoes, then employed in subsequent challenge studies [126–128]. A minute percentage of these selected filarial worms were shown to have reduced susceptibility to multiple ML doses following a single infection; hence these studies established that single high-dose MLs administered to microfilaremic dogs can exert meaningful selection pressure on an existing population of microfilariae. This pressure eliminated the most susceptible microfilariae rapidly and afforded the opportunity for less susceptible microfilariae to remain.

Efforts are underway to utilize longer-duration, continuous-acting ML formulations for heartworm disease prevention. Unfortunately, these long-acting and high-dose formulations offer a sustained duration of chemotherapeutic activity; however, while highly effective, their continuous systemic presence might limit potential immunotherapeutic stimulation or memory response.

Such selection pressure would be less likely to occur in dogs in endemic areas receiving short-acting monthly ML treatment, as new infections would be subjected to a direct, chemotherapeutically driven innate exposure effect as well as a host immune-memory-response effect. The repeated exposure and monthly clearance of new infective larvae present in the tissue of the dog could elicit a memory immune response similar to that seen in historic larval vaccination research, where *D. immitis* larvae were eliminated, [93], even when the complement-inhibiting capability of the filariae is intact.

Even in dogs already infected with heartworms, less selection pressure would be exerted by low-dose MLs than high-dose MLs. Well-controlled studies in dogs have shown that numerous monthly administrations of MLs can be necessary to eliminate microfilarial burdens [15]. As discussed in this review, a likely scenario is that low-dose MLs worked by initially exposing only a portion of the larval filarial worms to immune clearance. Subsequent monthly doses would affect portions of the remaining microfilarial population, eventually stimulating an immune response and amicrofilaremia. Amicrofilaremia is a known and documented immune-mediated phenomenon that occurs naturally in the individual host; therefore the concept that such a response would result from natural microfilarial vaccination or via vaccination, as a result of low-dose ML administration is logical.

It is further documented [85, 86, 129, 130] that repeated low-dose administration of an ML can also affect the reproductive capability and eventually the viability of the adult heartworms. Interestingly, in these studies this pronounced adulticidal effect is not seen as readily with high-dose monthly treatments, indicating this long-term response might be more immune mediated, than dose related.

Although preventive prophylaxis with MLs does not target microfilariae *per se*, research included in this literature review has demonstrated that microfilariae in fact represent the great potential for genetic variability seen in adult filarial worms. Therefore, maximizing the host immune response will likely play a larger role in the effective control of this genetically variable population than utilization of higher or longer acting doses of MLs.

## Conclusions

A review of the literature provides new insight into the way in which MLs likely affect filarial worms and set the stage for the deaths of microfilariae and larvae. It appears that, rather than killing juvenile filariae as a result of direct action, MLs expose susceptible, drug-contacted microfilariae and filarial larvae to the host’s innate immune system, producing an immediate immune response that kills microfilariae and larvae. This explanation may account for the historic lack of apparent ML activity against microfilariae and filarial larvae in vitro. Thus, understanding and further leveraging the memory response, both humoral and cellular immunity, of the host to worms exposed by MLs to the immune system could be the most important consideration in ML-driven filarial-disease management and prevention programs in humans and animals. An understanding of the parasite life-cycle is critical, as is a working knowledge of vaccinology. The host tissue compartment infected; the level of exposure to new infections; the dose of ML administered; the timing, duration, and frequency of dosing; and the host’s immunocompetence all appear to play roles in creating an active immune response in the infected individual and the resultant elimination of filarial worms. Filarial mass at the time of ML administration also has an impact on the immune response and potential antibody production. Should a significant antibody response to microfilariae be generated, the antibodies should be able to clear remaining and newly produced microfilariae, including those in the uterus of adult female worms; and could potentially affect the viability of the adult. When a significant antibody response to larval stages is generated, immune responses can be generated in turn, which can prevent or reduce new infections with filarial worms. Immune response and antibody production likely play critical roles in clearing larval infections as well as microfilarial burdens, including larvae and microfilariae that might be less susceptible to the effects of MLs. All options, including the concurrent use of vaccines should be considered to maintain filarial immunity and minimize filarial selection. Filarial diseases can affect the health and livelihood of humans and animals dramatically. Eliminating or controlling these diseases will only be possible with diligence and education. It is the hope of the author that the literature review and discussion presented here may act as an impetus for additional, targeted research to increase our understanding and provide more effective use of ML drugs in filarial infection prevention and control programs.

## Abbreviations

DEC: Diethylcarbamazine
ES/ES: Excretory-secretory
FCA: Freund’s complete adjuvant
GABA: Gamma-aminobutyric acid
GluCls: Glutamate-gated chloride ion channels
IFA: Indirect fluorescent antibody
IgG: Immunoglobulin G
IVM: Ivermectin
L1: First-stage larvae
L2: Second-stage larvae
L3: Third-stage larvae
L4: Fourth-stage larvae
ML/MLs: Macrocyclic lactones (includes avermectins and milbemycins)
